# Use of complementary and alternative medicine in patients with health complaints attributed to former dental amalgam fillings

**DOI:** 10.1186/s12906-016-0996-1

**Published:** 2016-01-22

**Authors:** Agnete E. Kristoffersen, Frauke Musial, Harald J. Hamre, Lars Björkman, Trine Stub, Anita Salamonsen, Terje Alræk

**Affiliations:** 1 The National Research Center in Complementary and Alternative Medicine, (NAFKAM), Department of Community Medicine, Faculty of Health Sciences, UiT The Arctic University of Norway, Tromsø, Norway; 2 Institute for Applied Epistemology and Medical Methodology at the University of Witten-Herdecke, Freiburg, Germany; 3 Dental Biomaterials Adverse Reaction Unit, Uni Research Health, Bergen, Norway

## Abstract

**Background:**

The dental filling material amalgam is generally well tolerated. However, a small proportion of dental patients experience health complaints which they attribute to amalgam. The symptom pattern is often similar to patients with medically unexplained physical symptoms (MUPS) and the health complaints may persist after amalgam removal. Among patients with MUPS, the use of complementary and alternative medicine (CAM) seems to be high. The aim of this survey was to describe the prevalence and range of CAM use among people with health complaints attributed to dental amalgam fillings in which the health problems persist after the removal of all amalgam fillings. Specific attention was paid to (1) self-reported effects of CAM, (2) differences in CAM use dependent on self-reported health, and (3) gender differences in self-reported CAM use.

**Methods:**

A survey was distributed to all members of The Norwegian dental patient association (NDPA) (*n* = 999), the response rate was 36.4 %. The anonymous questionnaire asked for socio-demographic data, health complaints related to former amalgam fillings, subjectively perceived health status, symptoms, and experience with therapeutic interventions, mostly from the spectrum of CAM. Only participants who had all their fillings removed, which was the vast majority, were analysed.

**Results:**

A total of 88.9 % of included respondents had used at least one CAM modality, with a higher proportion of men (95.7 %) compared to women (86.2 %, *p* = 0.015). The most frequently used therapies were dietary supplements, vitamins and minerals recommended by a therapist (used by 66.7 %) followed by self-prescribed dietary supplements, vitamins and minerals (59.0 %), homeopathy (54.0 %), acupuncture (48.8 %) and special diets (47.5 %). Use of CAM was similar for participants reporting normal to good health compared to participants reporting poor health. For all but two CAM modalities, the self-reported treatment effect was better in the group reporting normal to good health compared to the group reporting poor health.

**Conclusions:**

CAM was widely used by participants in our study, a finding similar to findings from studies of MUPS patients. To date, health problems associated with the use of dental amalgam is not an accepted diagnosis in the healthcare system. Consequently, people suffering from such complaints experience a lack of adequate treatment and support within conventional health care, which might have contributed to the high number of CAM users in this study.

## Background

Amalgam, an alloy of mercury and other metals, has been used as a dental filling material since the 19^th^ century and is well tolerated by most people [1]. However, a proportion of people experience health complaints, which they attribute to amalgam [2–4]. Moreover, for some of these patients the health complains persist even after removal of amalgam fillings. The question whether dental amalgam can cause general health complaints, apart from very rare cases of type-1 hypersensitivity, remains controversial [1]. Following removal of all dental amalgam fillings, an average of three-fourth of people with amalgam-attributed health complaints report improvement or recovery, while the remainder report no or little improvement or even deterioration [1] (deterioration rates are 0.5 %–2 % in population-based surveys [5, 6] and up to 15 % in selected samples [7–9]).

Health complaints persisting after amalgam removal are often similar to symptom patterns associated with medically unexplained symptoms (MUPS), such as fibromyalgia and myalgic encephalomyelitis (ME). Many of these patients use complementary and alternative medicine (CAM) [10–12]. A study of fibromyalgia patients in the U.S. found that 92.6 % of the participants (mostly women) reported to have used some kind of CAM [10]. CAM is also frequently used by patients with health complaints related to amalgam [9, 13–16]. In recent studies, the most commonly reported CAM modalities for this patient group were dietary supplements/vitamins (58–92 %) [9, 14, 15, 17], homeopathy (17–26 %) [9, 13, 14], acupuncture (13–28 %) [2, 9, 14, 16] and chiropractic (18–21 %) [14, 16]. In Norway the most commonly reported CAM modalities used for amalgam-related health complaints are dietary supplements, acupuncture and homeopathy (65 %, 28 % and 26 % respectively) [9, 17]. The literature has revealed that the prevalence and associations for use of CAM differs between men and women with regard to several socio demographic variables [18–23] and underline the importance of gender-specific analyses [24].

The dental amalgam safety issue has been debated since the 19^th^ century in the U.S [25], since the 1920s and 1930s in Germany [26, 27] and Denmark [28, 29], and since the 1970s internationally [30–35] with patient organizations active in a number of European countries, North America, Australia and New Zealand [25]. In Norway the debate started in the early 1980s [36, 37] and in 1990 *The Norwegian dental patient association* (NDPA, *Forbundet Tenner og Helse* in Norwegian) was founded. NDPA is a non-profit patient organization working for a non-toxic dentistry. The association also works to ensure that people, who experience themselves as being hurt/injured by dentistry, shall be entitled to rehabilitation and community support. The survey reported here was conducted in close cooperation with NDPA and is thus in accordance with the emphasis on patient involvement in recent health strategy documents from Norwegian health authorities.

In a representative survey of the adult Norwegian population from 2006, between 5 % and 8 % of the participants expressed the belief that their amalgam fillings had affected their health adversely. Further, a total of 43 % of adults with amalgam fillings had some or all amalgam fillings removed and in 8 % of these participants, the reasons for the removal of the fillings were exclusively due to general health concerns [5]. Moreover, knowledge about the use of CAM among Norwegians with health complaints attributed to amalgam, is limited to a few studies that describe a limited number of CAM modalities [9, 17].

This cross-sectional survey is part of a collaborative treatment project for people with suspected adverse effects from dental amalgam and serve as a basis for the development of a treatment program, especially designed for this group of patients. The overall aim of this study was to describe the prevalence and range of CAM use among people with health complaints attributed to amalgam fillings, in which these health complaints persist after the removal of all amalgam fillings. Specific attention was paid to (1) self-reported effects of CAM treatments on health complaints, (2) potential differences in CAM use between participants with self-reported good vs. poor health, and (3) possible gender differences in self-reported CAM use.

Since there is no well-established, general pathophysiological explanation for the experienced symptoms in this group, we will use the term “amalgam-attributed health complaints” throughout the manuscript to denote general symptoms or health complaints for which the people affected or other concerned persons suspect the cause to be amalgam fillings, regardless if such a causal association has been substantiated or not.

## Methods

The survey was distributed to all members of NDPA in December 2011 with a reminder in February 2012. No inquiry about medical diagnoses was made, thus, no information on whether the amalgam-attributed health complaints of the participants could be explained by specific diseases or were medically unexplained (MUPS) was possible. Common to all participants was the attribution of their health complaints to former dental amalgam fillings.

The study participants returned the questionnaires anonymously to The National Research Center in Complementary and Alternative Medicine (NAFKAM) by means of a pre-stamped envelope. The anonymous questionnaire included socio demographic data, conditions related to the amalgam removal, subjectively perceived health status, symptoms, and experience with therapeutic interventions, mostly from the spectrum of CAM. Participants with remaining amalgam fillings were not asked to complete the survey and were therefore excluded from the study.

In this study a participant was defined as a CAM user, according to his or her answer to the following question:

*If you have removed all your amalgam fillings because of health complaints, which other treatment modalities (forms) have you specifically tried for those health complaints? (Tick (x) for every modality you have tried or not tried. Specify the name of the medication, diets and treatment institutions you have tried.*

*Dietary supplements, vitamins and minerals recommended by therapist (DSVMT), Dietary supplements, vitamin and minerals self-prescribed (DSVMS), Homeopathy, Acupuncture, Special diet, Reflexology, Massage, Herbs, Healing, Ear Acupuncture, Kinesiology, Magnetic field therapy, Naprapathy, Biopathy, Thought field therapy, Rehabilitation in a CAM institution, Craniosacral therapy, Lightening process.*

Participants answering “*I have tried*” for at least one of the CAM modalities listed above were defined as users. Participants who answered “*I have not tried*” or had missing values in combination with no statement of effect for all listed CAM modalities were defined as non-users of CAM. The perceived effect of the treatment was indicated for each treatment as either “good effect”, “small/no effect” or “worsening”.

The CAM modalities were classified in accordance with the recommendation from The National Center for Complementary and Integrative Health (NCCIH) into the following five categories: 1) alternative medical systems, or complete systems of therapy and practice such as Traditional Chinese Medicine and homeopathy; 2) mind-body interventions, or techniques designed to facilitate the mind's effect on bodily functions and symptoms such as meditation; 3) biologically-based systems, including herbalism; 4) manipulative and body-based methods, such as chiropractic and massage therapy and 5) energy therapies such as healing [2].

Between-group differences were analyzed using chi-square tests for binary data analyzing one variable at the time and one-way ANOVA test for continuous data in SPSS for Windows (version 22.0, SPSS, Inc., Chicago, IL). Significance level was defined as *p* <0.05 without *p*-value adjustment for multiple comparisons.

The Norwegian Data Inspectorate has been notified about the study and the Regional Committee for Medical and Health Research Ethics (REK) has approved the study (REK reference 2011/1281).

## Results

**Fig. 1:**
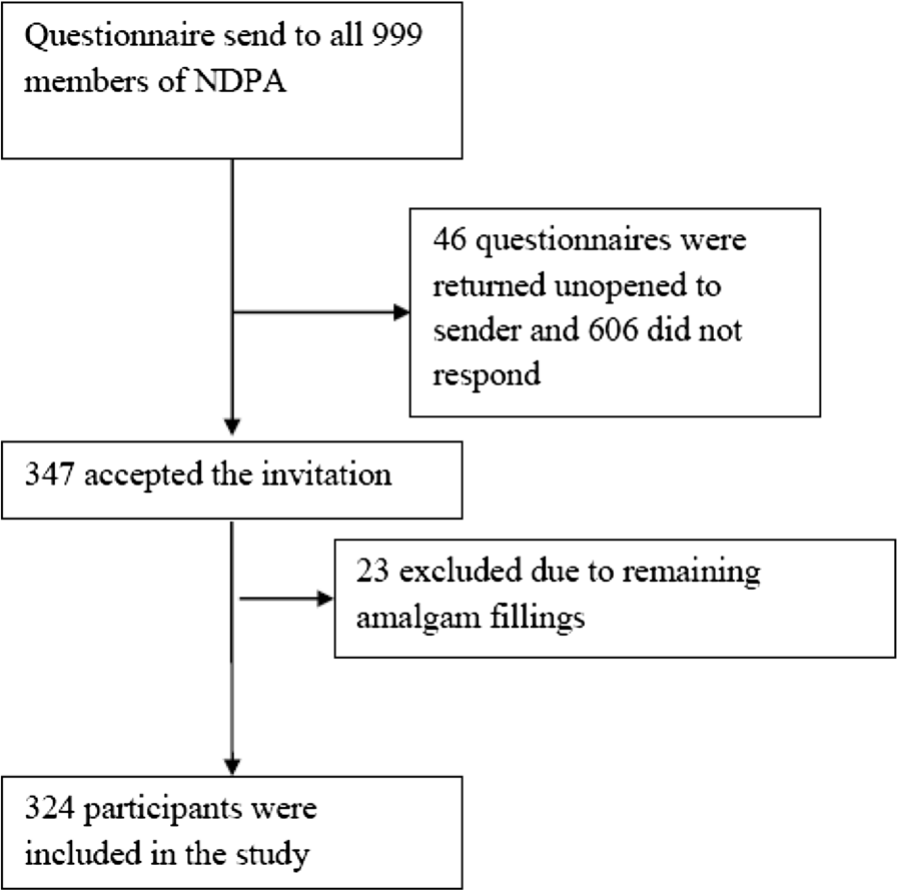
Flow chart showing the selection of the studied population. NDPA: Norwegian Dental Patients Association

A total of 999 envelopes with questionnaires were sent out, of which 46 were returned unopened to sender. Overall, 953 members of NDPA received the questionnaire and a total of 347 responded (36.4 % response rate) (Fig. 1).

### Basic characteristics of the participants

The majority of the participants were women (71.6 %) and most participants reported normal to good health (62.4 %). Half of the participants were holding a university degree and mean age was 60 years. The women were slightly older than the men (*p* = 0.001) and men were more often still working. No significant gender differences were found with regard to education nor self-reported health (Table 1).

**Table 1:** Basic characteristics of the participants

	Total	Men	Women	*p*-value
	% (*n*)	% (*n*)	% (*n*)	
Gender				
Women	71.6 (232)			
Men	28.4 (92)			
Age				
Mean	60.0	58.8 (SD 11.12)	61.3 (SD 10.26)	0.001*
Education				
Primary school	13.4 (42)	7.9 (7)	15.6 (35)	
Secondary school	27.4 (86)	32.6 (29)	25.3 (57)	
High school	15.6 (49)	13.5 (12)	16.4 (37)	
University, lower grade	22.0 (69)	25.8 (23)	20.4 (46)	
University, higher grade	27.1 (68)	20.2 (18)	22.2 (50)	0.252**
Self-reported health				
Normal to good	62.4 (199)	64.8 (59)	61.4 (140)	
Poor	37.6 (32)	35.2 (32)	38.6 (88)	0.568**
Working				
Yes	59.0 (128)	71.0 (49)	53.4 (79)	
No	41.0 (89)	29.0 (20)	46.6 (69)	0.014**

*One-way ANOVA test

**Pearson Chi-Square test

### CAM use and perceived effect of CAM in the total population

The mean number of different CAM therapy modalities used per participant was 5.7 with a median of 5 in both men and women, ranging from 0 to 15 modalities. The most commonly used CAM modalities were DSVMT (in 66.7 % of participants), DSVMS (59.0 %), homeopathy (54.0 %), acupuncture (48.8 %), reflexology (42.3 %), massage (40.1 %) and healing (33.6 %). The perceived effects of dietary supplements, vitamins and minerals (DSVMT/DSVMS) were mostly reported to be good (63.0–74.7 %). Roughly half of users reported good effect of homeopathy (57.4 %), reflexology (48.6 %) and massage (41.4 %). By contrast, only one third of users reported good effects of healing (33.8 %) and acupuncture (38.8 %). Of the studied population, 12.3 % (*n* = 40) reported worsening of symptoms that they related to their use of one or more CAM modalities. In seven out of 18 treatment modalities worsening of symptoms were reported by 5 % or more. Lightning process and thought field therapy were the only treatment modalities with no worsening of symptoms reported (Table 2).

**Table 2:** CAM use and perceived effect in the total population (*n* = 324)

	Reported use	Good effect	No effect	Worsening
	% (*n*)	% (*n* ^a^)	% (*n* ^a^)	% (*n* ^a^)
Over all CAM use	88.9 (288)			
Alternative medical systems	68.5 (222)			
Homeopathy	54.0 (175)	57.4 (81)	39.7 (56)	2.8 (4)
Acupuncture	48.8 (158)	38.8 (52)	58.2 (78)	3.0 (4)
Ear acupuncture	32.7 (106)	38.8 (33)	58.8 (50)	2.4 (2)
Mind-body interventions	16.0 (52)			
Thought field therapy	12.0 (39)	37.5 (12)	62.5 (20)	0 (0)
Lightning process	4.9 (16)	46.2 (6)	53.8 (7)	0 (0)
Biologically-based systems. including herbalism	84.3 (273)			
DSVMT	66.7 (216)	74.7 (139)	23.1 (43)	2.2 (4)
DSVMS	59.0 (191)	63.0 (92)	32.2 (47)	4.8 (7)
Special diet	47.5 (154)	74.4 (96)	23.3 (30)	2.3 (3)
Herbs	37.7 (122)	55.6 (50)	38.9 (35)	5.6 (5)
Biopathy^b^	13.3 (43)	40.6 (13)	56.3 (18)	3.1 (1)
Manipulative and body-based methods	61.4 (199)			
Reflexology	42.3 (137)	48.6 (52)	45.8 (49)	5.6 (6)
Massage	40.1 (130)	41.4 (41)	43.4 (43)	15.2 (15)
Kinesiology	29.3 (95)	43.3 (29)	50.7 (34)	6.0 (4)
Naprapathy^c^	13.3 (43)	46.4 (13)	46.4 (13)	7.1 (2)
Craniosacral therapy	8.6 (28)	42.9 (9)	47.6 (10)	9.5 (2)
Energy therapies	41.7 (135)			
Healing	33.6 (109)	33.8 (26)	63.6 (49)	2.6 (6)
Magnetic field therapy	19.1 (62)	29.5 (13)	61.4 (27)	9.1 (4)
Rehabilitation in a CAM institution	9.3 (30)	80.0 (20)	16.0 (4)	4.0 (1)

^a^Due to missing responses to the question about effect, the n regarding effect might be lower than for use of the CAM modality

^b^Biopathy is a treatment system that encompasses several different alternative diagnostic tools and therapies such as homeopathy, herbs, reflexology

^c^Naprapathy is a system of specific examination, diagnostics, manual treatment and rehabilitation of pain and dysfunction in the neuro-musculoskeletal system

### Gender specific CAM use and perceived effect of CAM

Overall CAM use was more frequently reported by men (95.7 %) than women (86.2 %) (*p* = 0.015). Among men, the most commonly used CAM modalities were DSVMT (72.8 %) followed by DSVMS (71.7 %), homeopathy (56.5 %) and special diet (55.4 %). Among women, DSVMT was most commonly used (64.2 %), followed by DSVMS (53.9 %), homeopathy (53.0 %) and acupuncture (48.7 %).

Comparing the use of individual CAM modalities between women and men, women were significantly more likely to use DSVMS (71.7 % vs. 53.9 %, *p* = 0.003) and less likely to use naprapathy (6.5 % vs. 15.9 %, *p* = 0.024). No significant gender differences were found in regard to CAM categories, though with a trend towards more frequent use of biological-based systems by men (*p* = 0.064).

No significant gender differences were found with regard to self-reported effect of the received CAM treatment (Table 3).

**Table 3:** Gender- and health specific use and effect of CAM

	Use of CAM			Good effect of CAM			Use of CAM			Good effect of CAM		
	Men	Women	*p*-value	Men	Women	*p*-value	Normal to good health	Poor health	*p*-value	Normal to good health	Poor health	*p*-value
	% (*n*)	% (*n*)		% (*n*)	% (*n*)		% (*n*)	% (*n*)		% (*n*)	% (*n*)	
Overall CAM use	95.7 (88)	86.2 (200)	0.015				89.9 (179)	88.3 (106)	0.650*			
Alternative medical systems	68.5 (63)	68.5 (159)	0.992				64.3 (128)	75.8 (91)	0.032*			
Homeopathy	56.5 (52)	53.0 (123)	0.568	52.3 (23)	59.8 (58)	0.403*	50.3 (100)	60.0 (72)	0.091*	68.4 (54)	42.4 (25)	0.002*
Acupuncture	48.9 (45)	48.7 (113)	0.973	33.3 (13)	41.1 (39)	0.405*	46.2 (92)	54.2 (65)	0.170*	49.3 (37)	25.9 (15)	0.006*
Ear acupuncture	31.5 (29)	33.2 (77)	0.851	32.0 (8)	41.7 (25)	0.405*	34.2 (68)	30.8 (37)	0.539*	43.4 (23)	29.0 (9)	0.191*
Mind-body interventions	14.1 (13)	16.8 (39)	0.553				15.1 (30)	18.3 (22)	0.445*			
Thoughtfield therapy	10.9 (10)	12.5 (29)	0.684	42.9 (3)	36.0 (9)	0.740*	12.6 (25)	11.7 (14)	0.813*	47.6 (10)	18.2 (2)	0.139**
Lightening process	4.3 (4)	5.2 (12)	0.757	33.3 (1)	50.0 (5)	1.000**	2.5 (5)	9.2 (11)	0.008*	60.0 (3)	37.5 (3)	0.592**
Biologically-based systems. including herbalism	90.2 (83)	81.9 (190)	0.064				86.9 (173)	80.8 (97)	0.143*			
DSVMT	72.8 (67)	64.2 (149)	0.139	74.2 (46)	75.0 (93)	0.905*	68.8 (137)	64.2 (77)	0.389*	80.7 (96)	63.6 (42)	0.011*
DSVMS	71.7 (66)	53.9 (125)	*0.003*	66.1 (37)	61.1 (55)	0.546*	60.3 (120)	58.3 (70)	0.729*	72.2 (70)	43.8 (21)	0.001*
Special diet	55.4 (51)	44.4 (103)	0.073	76.1 (35)	73.5 (61)	0.746*	42.7 (85)	56.7 (68)	0.016*	79.5 (58)	67.3 (37)	0.119*
Herbs	35.9 (33)	38.4 (89)	0.676	63.0 (17)	52.4 (33)	0.355*	35.7 (71)	41.7 (50)	0.286*	58.8 (30)	50.0 (19)	0.408*
Biopathy	10.9 (10)	14.2 (33)	0.422	50.0 (5)	36.4 (8)	0.699**	10.1 (20)	19.2 (23)	0.021*	35.7 (5)	44.4 (8)	0.618*
Manipulative and body-based methods	60.9 (56)	61.6 (143)	0.898				59.3 (118)	65.5 (79)	0.245*			
Reflexology	41.3 (38)	42.7 (99)	0.822	41.9 (13)	51.3 (39)	0.378*	40.2 (80)	45.8 (55)	0.324*	51.6 (32)	43.2 (19)	0.392*
Massage	33.7 (31)	42.7 (99)	0.137	58.3 (14)	36.0 (27)	0.053*	38.7 (77)	43.3 (52)	0.413*	46.3 (25)	36.4 (16)	0.321*
Kinesiology	29.3 (27)	29.3 (68)	0.995	40.0 (8)	44.7 (21)	0.723*	27.1 ((54)	33.3 (40)	0.240*	40.6 (13)	44.1 (15)	0.774*
Naprapathy	6.5 (6)	15.9 (37)	0.024	75.0 (3)	41.7 (10)	0.311**	12.1 (24)	14.2 (17)	0.586*	50.0 (8)	40.0 (4)	0.619**
Craniosacral therapy	5.4 (5)	9.9 (23)	0.196	80.0 (4)	31.3 (5)	0.119**	9.0 (18)	8.3 (10)	0.828*	75.0 (9)	0.0 (0)	0.001**
Energy therapies	40.2 (37)	42.2 (98)	0.739				40.7 (81)	44.2 (53)	0.544*			
Healing	35.9 (33)	32.8 (76)	0.593	37.5 (9)	32.1 (17)	0.641**	29.1 (58)	41.7 (50)	0.022*	47.2 (17)	20.0 (8)	0.012*
Magneticfield therapy	14.1 (13)	21.1 (49)	0.149	36.4 (4)	27.3 (9)	0.706**	22.6 (45)	14.2 (17)	0.065*	38.7 (12)	7.7 (1)	0.068**
Rehabilitation in a CAM institution	7.6 (7)	9.9 (23)	0.519	66.7 (4)	84.2 (16)	0.562**	10.6 (21)	7.5 (9)	0.366*	94.4 (17)	42.9 (3)	0.012**

*Pearson Chi-Square test

**Fisher's Exact test

### CAM use and perceived effect of CAM: subgroup analysis according to self-reported health

When men and women were divided into groups of self-reported health, no gender differences were found in the group reporting normal to good health. In the poor health group, on the other hand, the gender differences reported above remained, with the addition of another therapy, magnetic-field therapy, which was significantly more often used among women than men.

There were no significant differences in overall CAM use between the groups reporting normal to good health and poor health. However, the CAM category “alternative medical systems” was more commonly used among participants who reported poor health compared to participants who reported normal to good health (*p* = 0.032). With regard to individual CAM modalities, the poor health group was more likely to use special diets, spiritual healing, biopathy and Lightning Process than the group reporting normal to good health. The self-reported effects of the CAM therapy modalities were reported to be better in the normal to good health group than in the poor health group (Table 3).

## Discussion

In this cross-sectional survey of members of NDPA with persistent health complaints attributed to former amalgam fillings, 89 % had used CAM for their health complaints. DSVMT was the most commonly used CAM modality followed by DSVMS, homeopathy, acupuncture and special diet. Similar use was found in participants reporting normal to good health and participants reporting poor health. The self-reported effect of different CAM modalities was highest in the group with normal to good health. More men than women reported use of CAM in this study. Thus, similar to patients with MUPS, patients with amalgam attributed health complaints are frequent users of CAM.

Health care providers often find patients with medically unexplained symptoms difficult to handle, and misunderstandings between health care providers and patients seem to be common [38]. Since adverse effects from dental amalgam is not an accepted diagnosis in the healthcare system, the reasons why people with amalgam attributed health complaints turn to CAM modalities, as reported in our study, may be similar.

Our findings of high use of CAM in general and dietary supplements and vitamins/minerals in particular are in accordance with findings in other studies of patients with amalgam-attributes health complaints [9, 13–15, 17]. This high use might be partly caused by the fact that patient associations and some doctors and therapists recommend vitamins and minerals in conjunction with amalgam removal [39–41]. Our finding of rather frequent use of homeopathy was also reported in another Norwegian study of patients with amalgam-attributed health complaints [9]. The possible lack of adequate conventional treatment available for these health problems might be the reason for the high number of CAM users in this study. Also the fact that the period for CAM use in this study was “since onset of the health complaints” (instead of commonly “in the past year” or similar), might have contributed to the high frequency of CAM use.

The higher use of CAM among men than women in our study is not in accordance with findings in previous studies of other patient groups [18–22]. The reason for this might be the highly selected group of male participants due to membership in a patient association and removal of their amalgam filling at their own expense. The fact that more men than women were still working might have given more of the men the financially abilities to finance CAM use, since CAM is mainly paid out-of-pocket in Norway. The lower use of CAM among men than women in most other studies is often attributed to a presumption that men’s health care needs are better met within conventional health care [42], while we here see CAM use in a population that do not find their health care needs met within conventional health care [10]. Educational level and self-reported health were similar in men and women and these factors can therefore not explain the differences in CAM use.

The better self-reported effect of the CAM treatment among the participants with normal to good health compared to those with poor health is an interesting finding and not easy to explain. Generally, since this is a cross-sectional survey, it is impossible to make causal interpretations. Possibly, differential effects of CAM therapy could lead to different degrees of health improvement in participants with similar health status before therapy.

Worsening of symptoms following CAM treatment was reported by 12.3 % of the participants in our study. For homeopathy, worsening was reported by 2.8 % of users, which is much lower than in another Norwegian study where 26 % reported worsening after homeopathic treatment regardless of health complains [43]. The deterioration rate following CAM therapy for amalgam-attributed health complaints in this study (12.3 %) was similar to deterioration rates in three studies of amalgam removal for the same indication, also in highly selected patient groups (9.5 % of members of a Swedish dental patient association [7]; 14.7 % and 13 % of patients referred to dental material adverse reaction units in Sweden [29] and Norway [30], respectively) [7–9]. Possibly, deterioration following CAM treatment in this study could be related to characteristics of the selected patient group and not just to features of the CAM interventions.

### Limitations

The main limitation of this study is the highly selected target group: In order to identify and reach patients with persistent health complaints attributed to former amalgam fillings, subjects were recruited from a specific patient association and may therefore not be representative for the total patient group. A German study shows that members of fibromyalgia self-help groups use significantly more CAM than patients not affiliated with self-help groups [11] while in a Norwegian study of people with amalgam-attributed health complaints, those who had removed all their amalgam fillings were significantly more likely to use homeopathy and natural therapy than those who still had amalgam fillings [9]. The survey had a modest response rate (36.4 %) which may influence the generalizability of the findings. This survey did not contain diagnoses of the health complaints; therefore, the prevalence of related conditions such as MUPS cannot be assessed. Since the CAM use was not limited in time, but related to the amalgam health complaints, regardless of when they started, the recall period concerning CAM use might have been long and resulted in inaccuracies with regard to the reported use of CAM therapies. Also, the reported subgroup differences with regard to CAM use and self-reported CAM effects should be treated with caution, because of multiple hypothesis testing and due to low sample sizes in some subgroups.

### Interpretation

This is the first survey of CAM use among people with amalgam-attributed health complaints in Norway addressing a broad range of CAM modalities. To our knowledge, it is the first study of CAM use worldwide to focus on the subgroup of people with amalgam-attributed health complaints, in which the health complaints persist following complete amalgam removal, and is therefore a door opener to the field. The results from this study were used for the development of an Integrated Medical Care Rehabilitation program for this patient group, in which CAM is given as a part of the program.

## Conclusion

Findings from this study suggest that CAM was widely used by people with health complaints attributed to dental amalgam fillings, and who had removed all amalgam fillings, and were member of a patient organization, NDPA. The reasons for the considerably high use of various CAM modalities may be related to the experienced lack of support and treatment offers within the conventional health care system.

## Abbreviations

MUPS: Medically unexplained physical symptoms
ME: Myalgic encephalomyelitis
CAM: Complementary and Alternative Medicine
NAFKAM: National Research Center in Complementary and Alternative Medicine
OTC: Over the counter
NDPA: Norwegian Dental Patients Association (*Forbundet tenner og helse* in Norwegian)
NCCIH: National Center for Complementary and Integrative Health
DSVMT: Dietary supplements, vitamins and minerals recommended by therapist
DSVMS: Dietary supplements, vitamin and minerals self-prescribed
REK: Regional Committee for Medical and Health Research Ethics
